# Developmental intestinal microbiome alterations in canine fading puppy syndrome: a prospective observational study

**DOI:** 10.1038/s41522-021-00222-7

**Published:** 2021-06-23

**Authors:** Smadar Tal, Evgenii Tikhonov, Itamar Aroch, Lior Hefetz, Sondra Turjeman, Omry Koren, Sharon Kuzi

**Affiliations:** 1 The Hebrew University Veterinary Teaching Hospital and Koret School of Veterinary Medicine, Hebrew University of Jerusalem, Rehovot, Israel; 2 Azrieli Faculty of Medicine, Bar-Ilan University, Safed, Israel

**Keywords:** Microbiome, Clinical microbiology

## Abstract

Fading puppy syndrome (FPS) is a fatal condition in neonatal dogs. Intestinal microbial alterations, although never investigated, may be involved in its pathophysiology. The study examined the occurrence of FPS and its associations with dam, puppy, and husbandry characteristics, compared the intestinal microbial diversity of healthy puppies and those with FPS, and examined whether intestinal microbiomes are predictive of FPS. Day 1 and 8 post-partum (PP) rectal swabs were collected from healthy puppies and puppies which later developed FPS. Microbial compositional structure, including alpha and beta diversities and relative abundance of specific taxa were compared between groups, and microbial data was applied to a machine-learning model to assess the predictive performance of microbial indices of FPS or death. FPS occurred in 22/165 puppies (13%), with a 100% mortality rate. FPS was associated (*P* < 0.001) with decreased Day 1 PP puppy activity. Day 1 (*P* = 0.003) and 8 (*P* = 0.005) PP rectal beta diversities were different in puppies with FPS vs healthy ones. Increased Proteobacteria/Firmicutes ratio, increased relative abundance of Pasteurellaceae, and decreased relative abundance of Clostridia and *Enterococcus* were associated with FPS. A machine-learning model showed that Day 1 PP rectal microbiome composition accurately predicted FPS-related death. We found that specific rectal microbial phenotypes are associated with FPS, reflecting the significant role of microbiome alterations in this phenomenon. These findings may serve as useful microbial indices for early diagnosis of puppies at risk of FPS and may provide specific therapeutic targets.

## Introduction

Fading puppy syndrome (FPS) is a lethal condition in dogs, affecting up to 30% of litters up to 3 weeks of age, with most deaths occurring within the first seven days post-partum (PP)^1–4^. Its clinical presentation is non-specific (e.g., decreased nursing, progressive weakness, and death). Thus, establishing a definite diagnosis and providing successful specific therapy are generally unsuccessful, and mortality rates approach 100%^1,5,6^. Many etiologies of FPS have been suggested, including dystocia, bacterial and viral infections, failure of passive transfer of immunity, intestinal parasite infestation, inadequate maternal care, agalactia or poor milk quality, and other health, developmental, environmental, and husbandry factors^1,3,6–11^. Following dystocia, bacterial infections and septicemia are considered major causes of death in puppies, and are possibly the main etiology of FPS^3,12–21^. Bacterial infections often spread from dam to puppy through infected maternal secretions^16,17^, and translocation across the intestinal mucosal barrier may also induce systemic disease^18,22,23^. With sudden onset, non-specific symptoms, and rapid progression of neonatal septicemia^15,17,21,24^, its clinical management is extremely challenging, and prognosis is poor^3,20^.

Little progress has been made over the last decades towards improving the treatment and outcome of FPS. Although some FPS-related deaths may involve bacterial infections, their pathogenesis, and the role of the host’s intestinal microbiome in FPS remain unclear. Current theories and data from preterm human infants suggest that neonatal microbial dynamics and composition disruption are associated with diseases, such as necrotizing enterocolitis (NEC) and late onset sepsis (The European Perinatal Health Report, <https://europeristat.com/images/doc/EPHR2010_w_disclaimer.pdf> 2010)^25^. Nevertheless, similar theories have not been investigated in FPS.

The mammalian intestinal tract contains diverse microbial populations, playing complex roles in the host’s health, providing nutritional substrates, modulating the immune system, and aiding in defense against intestinal pathogens^26,27^. Initial intestinal colonization in human newborns commences with exposure to maternal vaginal and fecal microbiota, and it is vital for normal intestinal development and function^28–31^. Microbiome-associated gestational alterations and their effects on human newborns were extensively investigated due to their potential implications on health and disease states, both early in life and later on^32–36^. Maternal health status, Cesarean birth, milk quality, antibiotic administration, and premature birth are all associated with abnormal neonatal microbiome development, and are potentially linked to various diseases (e.g., asthma, atopy, and obesity)^32,37–43^. Studies employing DNA sequencing methods to investigate the intestinal microbiome in neonatal dogs are scarce^44,45^. Comparable to human neonates and kittens^46,47^, one study revealed temporal instability and substantial inter-individual variability, with progressive increases in bacterial richness between 2, 21, and 56 days PP in dogs^44^. To our knowledge, studies investigating possible associations between maternal and neonatal microbial alterations and neonatal diseases in dogs are unavailable.

The aims of this prospective study were: (1) to investigate the occurrence of FPS in purebred dogs and identify FPS-associated clinical and environmental factors; (2) to characterize the neonatal rectal microbiome constitution during the first week PP; (3) to compare the rectal microbiome composition of healthy and ‘fading’ neonatal puppies; and (4) to examine if neonatal rectal microbiome composition PP is associated with, and predictive of, FPS. Our results confirm that FPS is associated with early rectal dysbiosis, predictive of FPS and mortality. Fading puppies had distinctive rectal beta diversity, a greater Proteobacteria/Firmicutes ratio, increased abundance of Pasteurellaceae and decreased Clostridia and *Enterococcus* abundance compared to healthy puppies. Abnormal microbial development in FPS shows similar characteristics to dysbiosis–associated human neonatal diseases such as NEC, carrying potential for beneficial diagnostic and therapeutic uses, alongside possible “one-health” implications across species.

## Results

### Animal cohort characteristics and FPS occurence

The study included 165 puppies, of small (*n* = 48), medium (*n* = 81), and large (*n* = 36) breeds, from 25 litters (Table 1). The median litter size was six puppies (range, 2–13). The median dam age was 4 years (range, 2–7). FPS was diagnosed in 22/165 puppies (13.3%), of 12/25 litters (48%). All dogs with FPS died, despite therapy, within 2–11 days PP (median, 3.5). With a mortality rate of 100% of puppies with FPS, the outcomes (i.e., FPS and death) were used interchangeably in our analyses. FPS was not associated with dam age, parity and breed, parturition season, kennel, occurrence of parturition abnormalities, litter size, and puppy sex, or Day-1 and Day-8 rectal temperature (RT). Occurrence of FPS in one puppy was not associated with higher risk for FPS in littermates. FPS occurred more frequently (*P* < 0.001) in puppies which showed low Day-1 PP activity scores.

**Table 1:** Demographic data of 25 dams and their litters, and occurrence of parturition abnormalities and fading puppy syndrome.

Parturition number	Kennel^a^	Season	Dam age (years)	Parity	Parturition abnormalities	Breed	Litter size	Puppies with FPS
1	1	Winter	5	3	Dystocia	Border collie	5	0
2	2	Winter	4	3	PL, LP	Shih Tzu	4	0
3	2	Winter	2	1	Resusc	Pembroke Welsh Corgi	6	1
4	2	Winter	2.5	1	–	Maltese	3	0
5	1	Winter	6	4	–	Australian shepherd	8	5
6	1	Winter	3.5	2	–	Australian shepherd	10	4
7	1	Winter	6	4	–	Australian shepherd	4	0
8	1	Winter	4	3	–	Jack Russell Terrier	5	1
9	3	Spring	NA	NA	–	American Pit bull terrier	9	1
10	4	Spring	2	1	–	Cane Corso	13	1
11	2	Spring	2.5	2	–	Shih Tzu	8	0
12	2	Spring	4	3	–	CKCS	5	0
13	2	Spring	2.5	1	–	German shepherd	7	2
14	2	Spring	5	2	–	Shih Tzu	5	1
15	2	Summer	3	2	–	Caucasian shepherd	6	0
16	2	Summer	7	3	–	Shih Tzu	2	0
17	1	Autumn	5	4	–	Border collie	8	1
18	1	Autumn	4.5	3	–	Jack Russell terrier	6	1
19	1	Autumn	3.5	2	–	Australian shepherd	6	1
20	1	Winter	6	4	–	Australian shepherd	10	3
21	1	Winter	6	3	–	Australian shepherd	8	0
22	1	Winter	3	1	Dystocia, CS	Bernese Mountain Dog	10	0
23	2	Spring	3	2	–	Shih Tzu	5	0
24	1	Spring	4.5	3	–	Australian shepherd	7	0
25	1	Spring	5	3	–	Shetland sheepdog	5	0

*NA* not available, *PL* prolonged labor, *LP* large puppies, *Resusc* two puppies required resuscitation during parturition, *CS* Cesarean section, *CKCS* Cavalier King Charles spaniel.

^a^1 and 2 commercial breeding kennels, 3 municipal kennel, 4 unprofessional breeder.

Gross necropsy findings were available for 9/22 puppies with FPS and included diffusely hyperemic intestinal mucosa (6 puppies) and congested, hyperemic caudal lung lobes (1), while in the remaining two, no abnormal findings were noted.

### Microbial composition of rectal bacteria

**Fig. 1:**
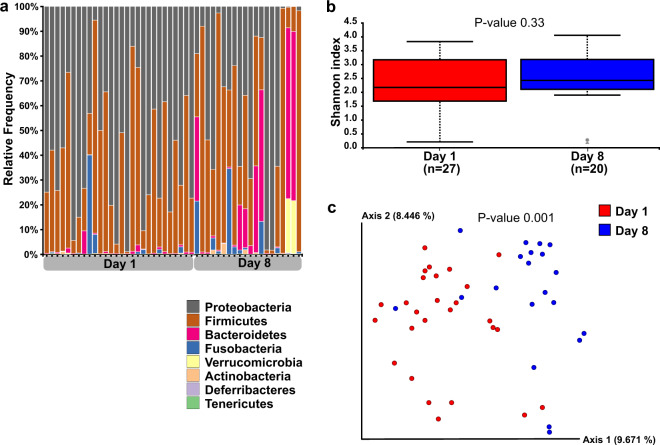
Rectal bacterial composition in healthy neonatal puppies on Days 1 and 8 post-partum. **a** Taxonomy bar plot; **b** alpha diversity analysis using Shannon index; **c** principal coordinate analysis plot representing beta diversity based on Jaccard distances (permutational multivariate analysis of variance, *P* = 0.001).

**Fig. 2:**
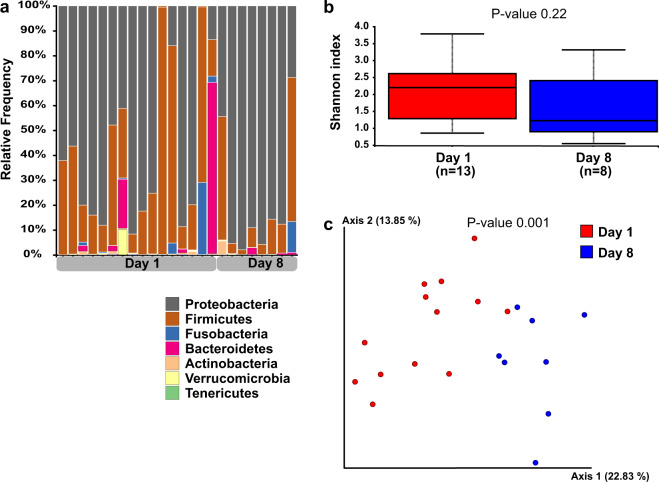
Rectal bacterial composition in puppies with Fading Puppy Syndrome on Days 1 and 8 post-partum. **a** Taxonomy bar plot; **b** alpha diversity analysis using Shannon index; **c** principal coordinate analysis plot representing beta diversity based on Jaccard distances (permutational multivariate analysis of variance, *P* = 0.001).

**Fig. 3:**
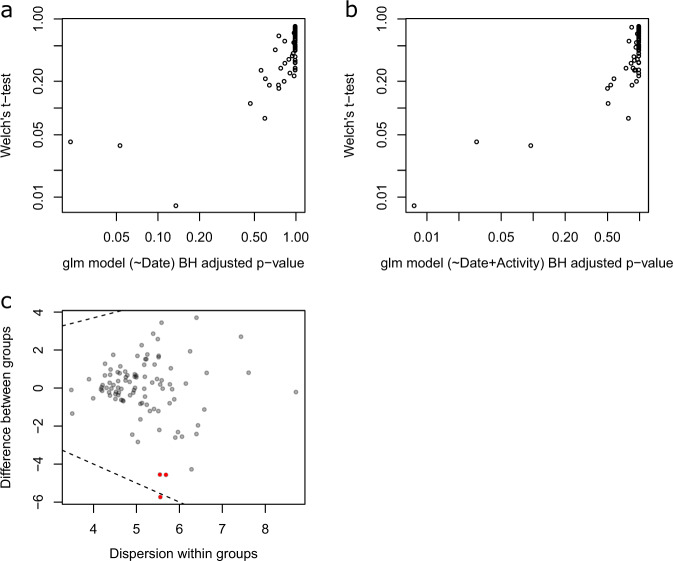
Differential abundance analysis (ALDEx2) of rectal microbiota in healthy neonatal puppies on Days 1 and 8 post-partum. Comparing the glm and *t*-test. Plots of the expected *P* values for variables output from the glm function with model. **a** Day postpartum as a fixed predictor and model; **b** with activity scale as a random effect predictor compared to the *P* values output by the Welch’s *t*-test. All values are the expected value of the test statistic after correction for multiple hypothesis testing; **c** effect size plot indicating between group difference (*Y* axis) and within group dispersion (*X* axis). Red represents features called as differentially abundant with p.adj < 0.1. Puppy ID was included as a random factor in all models. See Supplementary Table 2 for taxa information.

**Fig. 4:**
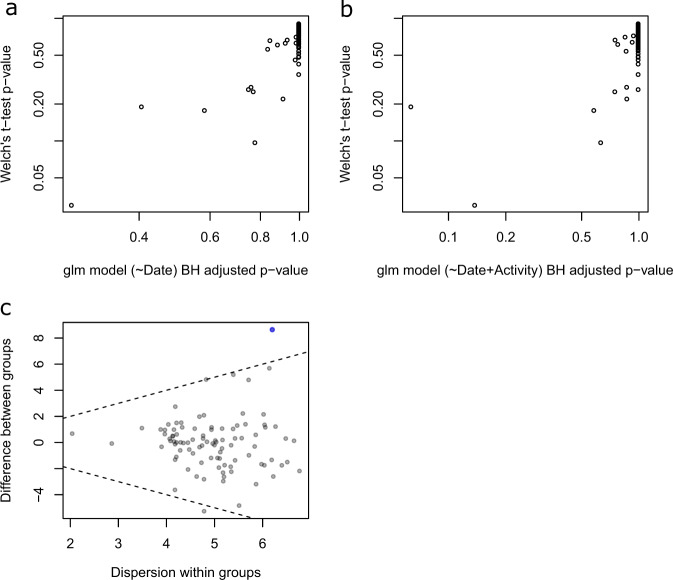
Differential abundance analysis (ALDEx2) of rectal microbiota in FPS puppies on Days 1 and 8 post-partum. Comparing the glm and *t*-test. Plots of the expected *P* values for variables output from the glm function with model. **a** Day postpartum as a fixed predictor and model; **b** with activity scale as a random effect predictor compared to the *P* values output by the Welch’s *t*-test. All values are the expected value of the test statistic after correction for multiple hypothesis testing; **c** effect size plot indicating between group difference (*Y* axis) and within group dispersion (*X* axis). Blue represents features called as differentially abundant with p.adj < 0.1. Puppy ID was included as a random factor in all models. See Supplementary Table 3 for taxa information.

**Fig. 5:**
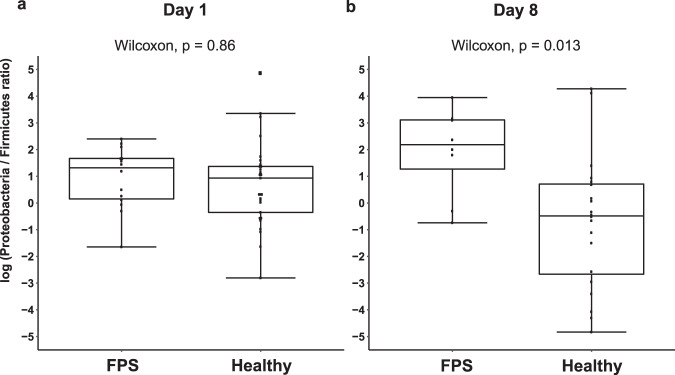
Rectal Proteobacteria/Firmicutes ratio in healthy puppies and those that later developed FPS. The Proteobacteria/Firmicutes ratio was calculated for both healthy puppies and those that later developed FPS in samples from **a** 1 and **b** 8 days post-partum. The *Y* axis indicates log transformed Proteobacteria/Firmicutes ratios. The rectal Proteobacteria/Firmicutes ratio on Day 8 was higher (*P* = 0.013) in puppies that later developed FPS compared to healthy puppies. Each box includes the interquartile range; the line within the box and whiskers represents the median and range, respectively. Each dot represents an individual sample. Sample size: Day 1: FPS-13, healty-27; Day 8: FPS-8, healthy-20.

Microbial composition analysis was performed on 93 rectal swabs, from 63 puppies, detailed in Supplementary Table 1. Taxonomy analysis was performed to characterize the rectal bacterial composition of healthy puppies (Fig. 1) and those with FPS (Fig. 2) within their first 8 days PP. Proteobacteria and Firmicutes phyla were the most dominant taxa in the majority of both Day-1 and Day-8 rectal samples of both groups (Figs. 1a and 2a). Other phyla contributing to the rectal microbiome of healthy and fading neonates were similar, and included Bacteroidetes, Fusobacteria, Verrucomicrobia, Actinobacteria, Deferribacteres, and Tenericutes (Figs. 1a and 2a). In healthy puppies, the relative abundance of genus *Epulopiscium* and of two species of *Clostridium* (*C. celatum* and *C. perfringens*) was significantly higher in Day-1 compared to Day-8 PP (Fig. 3 and Supplementary Table 2). In puppies with FPS, the relative abundance of bacterial genus *Streptococcus* was significantly lower in Day-1 compared to Day-8 PP (Fig. 4 and Supplementary Table 3). In healthy and fading puppies, alpha diversity (individual sample diversity, Figs. 1b and 2b) did not differ (*P* = 0.33 and *P* = 0.22, respectively) between Days 1 and 8 PP. Beta diversity (between sample diversity) was different (*P* = 0.001 for both groups) between Days 1 and 8 PP (Figs. 1c and 2c). In fading puppies, the Proteobacteria/Firmicutes ratio was significantly higher in Day-8 compared to healthy puppies (*P* = 0.013, Fig. 5).

### Predicting FPS occurrence based on rectal microbiome

**Fig. 6:**
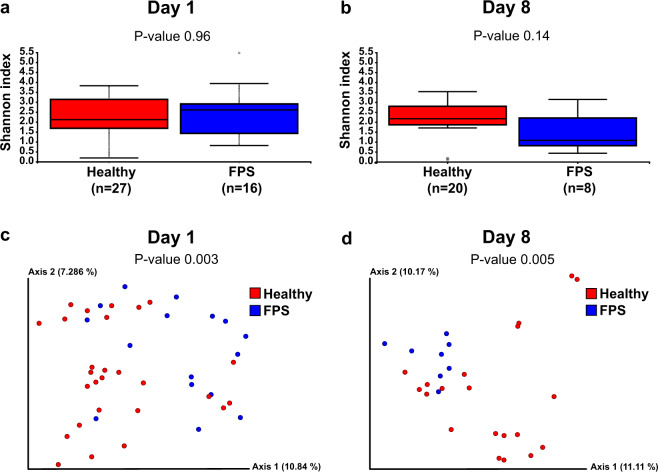
Rectal bacterial alpha and beta diversity in Days 1 and 8 post-partum samples from healthy puppies and those that sustained FPS later on. **a**, **b** alpha diversity analysis using Shannon index: the rectal microbial alpha diversity on Days **a** 1 and **b** 8 post-partum did not differ between healthy puppies and those that later sustained FPS (and died) using the Wilcoxon sum-rank test, *P* = 0.96 and *P* = 0.14, respectively). **c**, **d** principal coordinate analysis plots demonstrating beta diversity of healthy puppies (red) and those that sustained FPS later on (blue), which revealed significantly different rectal bacterial community composition in puppies that later sustained FPS compared to healthy puppies, both on **c** Day 1 (permutational multivariate analysis of variance, *P* = 0.003) and **d** Day 8 (permutational multivariate analysis of variance, *P* = 0.005) PP.

Diversity analyses were performed to examine potential rectal microbiome differences between healthy and ‘fading’ puppies within their first 8 days of life (Fig. 6). Alpha diversity did not differ between puppies which later succumbed to FPS and healthy ones on Days 1 (*P* = 0.96) or 8 (*P* = 0.14) PP (Fig. 6a, b). Beta diversity analyses revealed significant rectal microbiome differences between puppies which later developed FPS compared to healthy puppies on Days 1 (*P* = 0.003) and 8 (*P* = 0.005) PP (Fig. 6c, d).

**Fig. 7:**
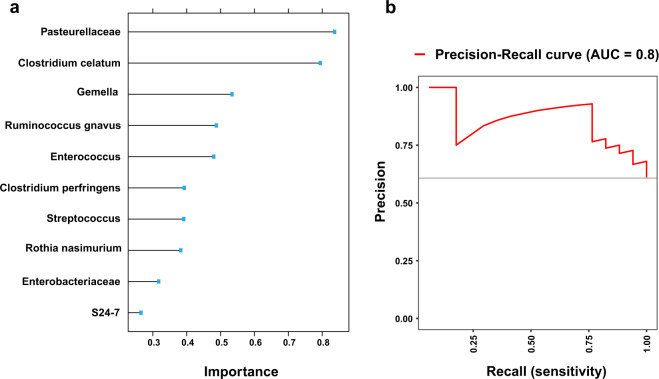
Prediction model of the outcome of neonatal puppies based on the rectal microbiome of healthy puppies and puppies that later succumbed to FPS on Day 1 post-partum. **a** Top 10 most predictive (important) bacterial taxa for the model performance; **b**Precision-Recall curve, showing the actual model predictive performance (AUC = 0.8).

In all, 40 puppies (healthy, 27; FPS, 13) examined on Day-1 PP, and 28 (healthy, 20; FPS, 8) examined on Day-8 PP served as test cohorts (leave-one-out cross-validation), using a supervised machine learning approach, to assess the rectal microbiome predictive performance of FPS occurrence; activity score was also included as a predictor. Day-1, but not Day-8 PP, rectal microbiome composition was highly predictive of FPS, and hence, of death (Precision-Recall AUC: 0.8, Fig. 7). The 10 most important taxa are described in Supplementary Table 4.

There were no correlations between rectal bacterial diversity of puppies with any dam or puppy parameters (e.g., dam age or body condition, and puppy RT and body weight (BW)).

## Discussion

Fading puppy syndrome is highly fatal, leading to neonate loss despite intensive supportive treatment. Although quite common, its pathophysiology is poorly understood^1,5,6^. The present study has investigated the rectal microbiomes of puppies which later developed FPS compared to healthy ones using rectal swabs. There was an association of rectal microbiota composition with FPS, and hence, with mortality. Specifically, Day 1 PP rectal bacterial beta diversity was significantly associated with occurrence of FPS. This finding is suggestive of an early dysbiosis profile, distinctive of fading puppies, characterized by a higher Proteobacteria/Firmicutes ratio, increased relative abundance of Pasteurellaceae and lower relative Clostridia and *Enterococcus* abundance. Day-1 PP rectal microbial indices were highly predictive of later FPS and may be useful for early detection and prognostication of puppies at risk of FPS and death. These present findings also suggest that intestinal microbial alterations may serve as potential therapeutic targets in fading puppies.

In agreement with a previous canine study^44^, Proteobacteria and Firmicutes were the most dominant phyla in most rectal samples for both Days 1 and 8 PP, supporting the role of these phyla as the most abundant early intestinal microbiome colonizers in neonatal dogs. The “early colonization” theory hypothesizes that these microbial colonies are acquired through direct contact with maternal and environmental bacteria during birth^30,48^. In humans, vaginal birth leads to a neonatal intestinal microbial population dominated by maternal birth canal and fecal microbial constituents (e.g., *Lactobacillus* spp. and *Bifidobacterium* spp.), whereas Cesarean section results in neonatal microbial population dominated by maternal skin microbial constituents (e.g., *Staphylococcus* spp.)^32^. In neonatal dogs delivered vaginally, the meconial microbial population composition resembled its maternal vaginal counterpart, while in neonates delivered by Cesarian section, it resembled both vaginal and oral maternal bacterial composition^49^. In the present cohort, with vaginal birth occurring in 24/25 parturitions, delivery mode was an unlikely confounder of results. Additional factors, most importantly nutrition, affect intestinal microbiota development;^50–52^ Nevertheless, all puppies herein fed solely by nursing from their dams, thereby excluding direct dietary impact on their Day-1 PP intestinal microbial composition.

Overall, early bacterial intestinal colonization studies in humans, dogs, and cats demonstrate deep inter-individual and temporal microbial variation, while the early intra-individual taxonomic diversity is limited, reflecting low alpha-diversity^44,47,48,53,54^. The latter is reasonable, as the immense diversity and complex structure of the symbiotic host mucosal–bacterial unit develops over time^48,53,54^. Similarly, herein, puppies showed relatively low individual bacterial (alpha) diversity that did not significantly increase at Day-8 PP. Concurrently, in agreement with previous studies^44,47,48,53,54^, puppies showed high inter-individual bacterial composition variability amongst group members, evident by the high dispersion of samples in the beta diversity principal coordinate space.

Interestingly, puppies which later died of FPS showed distinctive microbiomes, which were different compared to healthy puppies, as early as Day-1 PP, suggestive of characteristic rectal bacterial dysbiosis phenotypes, occurring in fading puppies. Specifically, Pasteurellaceae family members were important (Fig. 7) in fading puppies, possibly serving a future marker of FPS in microbial-based diagnostic tests. In a bacterial, culture-based study of dogs, *Pasteurella* spp. were mostly present in maternal oral mucosa, with lower proportions in maternal vaginal mucosa and neonatal meconium samples^49^. Despite methodical differences, the present results are non-comparable; higher rectal relative abundance of possibly orally–derived Pasteurellaceae in puppies which later developed FPS may suggest aberrant bacterial acquisition and colonization patterns in such puppies. Use of rectal swabs to collect samples possibly resulted in contamination with skin-derived bacteria from the anal area, including bacteria originating from dams’ oral mucosa, as dams lick their puppies. However, all puppies were exposed to the same maternal care (based on breeder reports); therefore, this possible source for Pasteurellaceae would not explain its higher abundance in fading puppies. The latter may also imply failure of upper-gastrointestinal-tract defense mechanisms (i.e., gastric acid and chloride secretion)^55^, possibly allowing abnormal intestinal bacterial colonization in fading puppies. Finally, Pasteurellaceae and other oral commensal bacteria were enriched in fecal microbial communities of human inflammatory bowel disease patients, and this abnormal bacterial localization was associated with gastrointestinal inflammation in mouse models^56,57^. Intestinal inflammation was previously reported in FPS^58^, and hyperemic intestinal mucosa was macroscopically evident in several puppies with FPS herein; thus, investigating possible associations between rectal Pasteurellaceae abundance and gross and histological intestinal abnormalities in fading puppies is warranted in future larger cohorts and may serve as a therapeutic target.

In contrast to Pasteurellaceae, Clostridia and *Enterococcus* were relatively more abundant in rectal microbiomes of healthy puppies. In a previous study in canine neonates, Clostridia comprised nearly 10% of identified sequences in 2-day-old puppies, whereas older ones (aged ≥ 3 weeks) showed decreased Clostridial abundance^44^. Similarly, Clostridia were relatively more abundant in Day-1 PP microbiomes than Day-8 PP microbiomes in this study and in healthy puppies at Day-1 PP than fading puppies, suggesting that *Clostridium* spp. may have beneficial roles in early neonatal health. For example, several Clostridia (e.g., *Clostridium* clusters XIVa and IV) produce butyrate, an important energy source for colonocytes^59^. In addition, early bacterial intestinal tract colonization is crucial for developing defenses against pathogenic bacteria, through direct competition and other inhibitory mechanisms^60^. A recent study in puppies demonstrated increased abundance of *Clostridium difficile*, but neither toxins A nor B were detected, suggesting early colonization with non-toxigenic *C. difficile*^45^. Similarly, in the same study, *Clostridium perfringens* was abundant, but without the presence of the virulent NetF toxin^45^. Therefore, decreased Clostridial abundance in puppies which later developed FPS possibly allows intestinal colonization by pathogenic bacteria, which potentially play a role in the pathogenesis of FPS. If this is true, microbial-based therapy, focused on restoring beneficial Clostridia in neonates at risk of FPS is warranted.

The apparent importance of *Enterococcus* in the early rectal microbiome of healthy puppies is less clear. While *Enterococcus faecium* is considered a beneficial probiotic in dogs^61^, it was also reported to increase in diarrheic dogs and was associated with hypocobalaminemia in healthy dogs^62,63^. In preterm babies, *Enterococcus* dominance was reported in extremely premature infants and it was associated with delayed normal microbiota development, but not with health or NEC^64,65^. Further studies are needed to better characterize *Enterococcus* specific strains in canine neonates and their possible beneficial or probiotic effects.

Relative decreased abundance of Clostridia, a member of the Firmicutes phylum, may have contributed to the increased Proteobacteria/Firmicutes ratio in rectal microbiomes of fading puppies herein. Remarkably, similar increased ratios are reported in human infants with NEC, a disease preceded by fecal dysbiosis^66^. Thus, microbiome optimization may provide a therapeutic strategy for preventing neonatal diseases that are associated with characteristic dysbiosis across species, carrying global, one-health implications.

The mechanisms leading to altered rectal microbial composition and their possible causal role in FPS are unknown. Although neonatal intestinal microbial acquisition depends on early contact with maternal and environmental microorganisms^30,48^, selective neonatal intestinal environmental conditions restrict microbial colonization^67,68^. This may be evident by the fact that although maternal intestinal microbial dysbiosis characterizes the third trimester of pregnency in women, such profiles are usually absent in neonatal babies^35^, suggesting complex bacterial-host immunologic signaling pathway interactions, resulting in immune tolerance and niche colonization^68^. FPS may involve local failure of defense mechanisms, allowing abnormal microbial colonization patterns, as demonstrated herein, or an initial primary “dysbiosis” may later lead to FPS, or both. Nevertheless, Day-1 PP rectal microbiome indices were highly predictive of later FPS (and death) when applied to a machine-learning model herein, supporting their role in FPS pathophysiology. Although the clinical application of these data is beyond the scope of this study, its results are promising, laying ground for future diagnostic microbiota-driven approaches to identify puppies at high risk of FPS, promoting preemptive therapeutic intervention and possibly decreasing FPS-associated mortality.

The present frequency of FPS and its resulting death rate are similar to previous reports (8.3–30% of litters)^58,69,70^. Low Day-1 puppy activity, although subjectively assessed, was positively associated with FPS. Though general and non-specific, its early indication of later FPS warrants intensive monitoring and support of such puppies by breeders.

This study has several limitations. Firstly, cohort size is limited, rendering it somewhat statistically underpowered. Nevertheless, present findings (e.g., limited Day 1 PP intra-individual bacterial diversity with a high inter-individual bacterial variability in neonates) are in line with previous results^44,47,48,53,54^, strengthening the current conclusions. Further, presence of a statistical and biological signal, despite litter-specific, breed-specific, and kennel-specific microbial signatures, lends strength to our findings. Secondly, maternal oral, intestinal, vaginal, milk and skin microbiota, which are major neonatal microbial acquisition sources^30,39,50^, were not investigated herein, precluding assessing their involvement in FPS. Nevertheless, healthy and fading littermates were exposed to identical maternal and environmental conditions, suggesting that additional, individual factors impact the intricate interactions between the early microbial colonizers and the puppies’ intestinal and immunological responses. Lastly, although dead puppies were grossly examined for abnormalities, necropsy, tissue bacterial cultures and fecal tests for specific enteropathogens of puppies that died of FPS herein were generally not performed, precluding determination of specific death causes. Overcoming these limitations in future studies warrants larger cohorts and including dam oral, vaginal, milk and skin samples for microbial analyses. Puppies with FPS that die should undergo comprehensive necropsy, with internal organ and blood bacterial culturing.

In conclusion, FPS occurs commonly in purebred dogs and is highly fatal. Microbial differences appear as early as Day-1 PP in puppies which later develop FPS, characterized by a distinctive rectal bacterial phenotype suggestive of dysbiosis. This finding supports the crucial role of intestinal microbiome patterned development in neonatal host health, and the pathogenesis of FPS. Additionally, Day-1 PP rectal microbial compositional structure is predictive of later FPS, suggesting potential use of microbial-based diagnostic tools, which will potentially promote early detection of puppies at risk of FPS, and therefore, require intensive monitoring and early intervention, both supportive, and aimed at microbial compositional restoration. Additional longitudinal studies, evaluating dam and puppy microbiomes and metabolomes, are needed to characterize the origin, structure, and functional characteristics of the canine neonatal intestinal microbiota in health and disease. Finally, similarities in dysbiotic rectal compositional phenotypes, may suggest a shared pathophysiology between human and canine neonatal diseases (i.e., NEC) that are associated with early dysbiosis, warranting further investigation.

## Methods

### Study design, dogs, and data collection

This prospective, observational study was approved by the institutional ethical committee (approval # KSVM-VTH/18_2017) and conducted during years 2018 to 2019. The study included pure-bred dams, whelping naturally or by Cesarean delivery, and their puppies, recruited with their owners’ signed consent. Dams were excluded if antibiotics were administered within 12 months prepartum, ensuring recovery of the microbiome to its pre-antibiotic treatment composition and excluding any effect on the intestinal microbial composition of their puppies^71^. Stillborn puppies, those that died within 24 h PP, those fed with milk replacer prior to the occurrence of FPS, or those treated with antibiotics prior to rectal swabbing (in order to avoid confounding effects of nutrition and antibiotics on the microbiome) were excluded^50,51,72^. Puppies with congenital defects at gross necropsy (i.e., cleft palate) and those with obvious non-FPS conditions (i.e., trauma), were also excluded.

Dams were routinely and currently vaccinated and dewormed and fed ad libitum balanced commercial canned and dry diets, suitable for pregnant and nursing dogs, at their breeders’ discretion. Collected dam history and husbandry information included age, breed, parity, fate of previous parturitions and litters, previous diseases, litter size, parturition data (e.g., length, occurrence of dystocia, stillborn puppies, and PP complications), and maternal litter care quality (e.g., licking, stimulating, and cleaning fecal and urinary secretions, time spent with the litter, and cannibalism). The season of parturition was recorded as follows: winter (December–February), spring (March–May), summer (June–August), and autumn (September–November). Dog breeds were divided based on the dams’ BW into small (BW < 1–10 kg), medium (BW, 10–25 kg), and large (BW > 25 kg) breeds. The sires’ ages and numbers of historical litters were recorded as well.

Recruited neonatal puppies underwent physical examination, including vital signs, general attitude, weight, sex, and abnormal findings. Their general attitude was assessed subjectively using an “activity scale score” as follows: score of 0, (“low activity”), quiet, with little or no movement, and no resistance to RT measurements; score of 1 (“moderate activity”), alert, showing movement, softly vocal and resisted RT measurements; score of 2 (“normal activity”), active, vocal resisting and attempting to escape upon RT measurement. Because neonates typically do not defecate, rectal swabs, rather than feces, were taken on days 1 and 8 PP, using sterile swabs (Copan, Brescia, Italy). Swabs were transported on dry ice to the laboratory within 2 h of collection and stored at −80°C for up to one year pending microbial analysis. Puppies were identified with color-coded, numbered collars. Rectal swabs were pre-marked with puppy number and sample time-point (i.e., Day-1 or Day-8 PP). After observing puppy behavior, samples were taken by gently inserting a swab rectally, followed by RT measurement. A new thermometer was used for each litter, which was disinfected and dried between littermates. Puppies were then weighed and physically examined, using the same scale, covered by a fresh paper towel, which was calibrated, and tared prior to each weighing.

### FPS diagnostic criteria, treatment, and outcome

Apparently healthy neonates on parturition day (i.e., observed nursing and alert) that progressively deteriorated, exhibiting weakness, decreased nursing, hypothermia (RT < 35.0 °C), dehydration, hypoglycemia (blood glucose < 90 mg/dL; measured by a glucometer [ACCU-CHEK® Performa]), or other clinical abnormalities (i.e., respiratory or gastrointestinal signs) within the first 2 weeks PP, were diagnosed with FPS.

Puppies with FPS received supportive treatment, including feeding tube, sublingual, or intravenous correction of hypoglycemia (blood glucose monitored q8h), and nutritional support with tube feeding milk replacer (Royal Canin Babydog Milk, 40 mL/kg per feeding). Hydration status was assessed clinically as well as by post-feeding urine specific gravity (USG) measurement. With dehydration (USG > 1.030), IV fluid replacement therapy was initiated (lactated Ringer’s solution, 3–6 mL/kg/hour for maintenance, after correcting dehydration). Hypothermia was treated by warming devices with monitored ambient temperature. Hypoxia (assessed by mucous membrane color) was treated with oxygen enriched environment. BW was monitored by repeated daily pre- and post-suckling weighing. Broad spectrum bactericidal antibiotics (amoxicillin-clavulanic acid, given IV [Clavenir, 1 g powder for solution, Laboratory Reig Jofre, Toledo, Spain] or PO [Maclivan, 400 mg/5 ml suspension, SANDOZ GmbH, AUSTRIA] at a dose of 15 mg/kg q12h) were prescribed to treat signs suggestive of infection, based on the attending clinician’s decision, as were canine probiotics (Pro-Kolin, Protexin Veterinary, Somerset, UK). Treatment was provided at the kennel or at the veterinary teaching hospital, based on the attending clinician’s assessment.

Puppies with FPS that died within seven days from onset of clinical signs were defined as non-survivors. When possible (depending on transport availability and time of death), puppies were submitted for necropsy within 2 h of death. Gross necropsy findings were recorded.

### Rectal microbiome analysis

Total DNA was extracted from swabs using PureLink microbiome DNA extraction kit (Invitrogen, Carlsbad, CA) following a 2-min bead-beating step^73^. Purified DNA was PCR amplified using PrimeSTAR Max (Takara-Clontech, Shiga, Japan) for the variable V4 region (using 515F-806R barcoded primers) of the 16S rRNA gene. Amplicons were purified using Agencourt AMPure XP magnetic beads (Beckman-Coulter, Brea, CA), and subsequently quantified using Quant-It Picogreen dsDNA quantitation kit (Invitrogen, Carlsbad, CA). Equimolar DNA amounts from individual samples were pooled and sequenced using the Illumina MiSeq platform at the Genomic Center, Azrieli Faculty of Medicine, Bar-Ilan University, Israel.

Sequencing data were processed using QIIME2 version 2019.4^74^. Single end sequences with similarity ≥99% were grouped into the same feature. Taxonomy was assigned using the GreenGenes database^75^. Chimeric sequences were removed with DADA2 (--p-trunc-len 185)^76^. Samples with <500 features, and features with total frequency <4, were filtered out. Rarefaction was done using 2649 sequences per sample (see Supplementary Fig. 1); while this depth likely does not represent all taxa in the samples, due to low DNA concentration in the samples, this threshold was used to maintain ample FPS samples for downstream analyses. Alpha and beta diversity were calculated based on rarefied datasets.

### Statistical analysis

The distribution pattern of quantitative variables was examined using the Shapiro–Wilk’s test. Quantitative variables were compared between groups (e.g., healthy vs. FPS) with the Student’s *t* test or Mann–Whitney (MW) *U*-test, for normally and non-normally distributed data, respectively. All tests were 2-tailed, and in all, *P* ≤ 0.05 was considered significant.

Bacterial alpha diversity was assessed by Shannon index. Statistical significance of microbial alpha diversity differences was confirmed using the Kruskal–Wallis test, followed by paired M-W tests, with Benjamini–Hochberg correction for false discovery rate. Beta diversity was calculated using Jaccard distances; when weighted and unweighted UniFrac distances were used, results were consistent (not shown). Statistical significance was confirmed using permutational multivariate analysis of variance (PERMANOVA).

Differences in relative abundances of bacterial taxa between groups were identified using the ANOVA-like differential expression tool (ALDEx2) (Figs. 3 and 4, Supplementary Tables 2 and 3). With non-normal data distribution, Spearman’s correlation tests were used to examine potential relationships between rectal bacterial diversity and quantitative dam, sire, and puppy parameters.

Microbial data from all rectal swab samples, along with activity scores, were used as “training and test cohort” for a machine-learning model (leave-one-out cross-validation). Then, remaining data were used as “test cohort” to assess if microbial indices were predictive of FPS. For the binary surviving prediction (i.e., Yes/No), a Random Forest Classifier algorithm from scikit-learn (version 0.19.1-3) was used, with a Precision-Recall area under curve (AUC) calculated to assess the predictive performance for occurrence of FPS and death^77^. Data were analyzed using R (R Core Team, 2019).

### Reporting summary

Further information on research design is available in the Nature Research Reporting Summary linked to this article.

## Supplementary information

Supplementary Information

Reporting Summary

## Data Availability

The datasets generated during the current study are available in EBI under accession number PRJEB42553 or in qiita (https://qiita.ucsd.edu/) under Study ID number 13428.

## References

[1] Davidson, A. P. *Recent Advances in Small Animal Reproduction* (eds. Concannon, P. W., England, G., Verstegen, III, J. & Linde-Forsberg, C.) (International Veterinary Information Service, Ithaca, 2003).

[2] Johnson, C. A. *Medicina Interna Del Cane E Del Gatto* 3rd edn (eds. Nelson, R. W., Couto, C. G.) Ch. 58 (Elsevier, Milano 2006).

[3] Veronsi, M. C. Neonatologia Veterinaria (eds. Veronesi, M. C., Castagnetti, C. & Taverne, M. A. M.) (EdiSES, 2013).

[4] Indrebø, A., Trangerud, C. & Moe, L. Canine neonatal mortality in four large breeds. *Acta Vet. Scand.* **49**, S2 (2007).

[5] Peterson, M. E. *Small Animal Pediatrics. The First 12 Month Of Life*. (eds. Peterson, M. E. & Kutzler, M. A.) Ch. 11 (Elsevier Saunders, 2011).

[6] Blunden T (2012). Fading puppies – reality or myth?. Practice.

[7] Creevy, K. E. *The Merck Veterinary Manual. Ch. Overview of Canine Herpesviral Infection* (Elsevier, 2013).

[8] Itoh N (2011). Prevalence of intestinal parasites and genotyping of Giardia intestinalis in pet shop puppies in east Japan. Vet. Parasitol..

[9] Hoskins JD (1999). Pediatric health care and management. Vet. Clin. North Am. Small Anim. Pract..

[10] Blunden AS, Hill CM, Brown BD, Morley CJ (1987). Lung surfactant composition in puppies dying of fading puppy complex. Res. Vet. Sci..

[11] Roth JA (1987). Possible association of thymus dysfunction with fading syndromes in puppies and kittens. Vet. Clin. North Am. Small Anim. Pract..

[12] Moon PF, Erb HN, Ludders JW, Gleed RD, Pascoe PJ (2000). Perioperative risk factors for puppies delivered by cesarean section in the United States and Canada. J. Am. Anim. Hosp. Assoc..

[13] Moon-Massat PF, Erb HN (2002). Perioperative factors associated with puppy vigor after delivery by cesarean section. J. Am. Anim. Hosp. Assoc..

[14] Daniel, J. & Spencer, E. *Small Animal Pediatrics. The First 12 Month Of Life* (eds. Peterson M. E., Kutzler M. A.) Ch. 15 (Elsevier Saunders, 2011).

[15] Munnich A, Grussel T, Leopold T (1995). [Experiences in diagnosis and therapy of puppy diseases in the first days of life]. Tierarztl. Prax..

[16] Munnich A, Lubke-Becker A (2004). Escherichia coli infections in newborn puppies–clinical and epidemiological investigations. Theriogenology.

[17] Schafer-Somi S, Spergser J, Breitenfellner J, Aurich JE (2003). Bacteriological status of canine milk and septicaemia in neonatal puppies–a retrospective study. J. Vet. Med. B Infect. Dis. Vet. Public Health.

[18] Go LL (1994). Quantitative and morphologic analysis of bacterial translocation in neonates. Arch. Surg..

[19] Askaa J, Jacobsen KB, Sorensen M (1978). Neonatal infections in puppies caused by Escherichia coli serogroups 04 and 025. Nord Vet. Med..

[20] Hoskins, J. D. (ed) *Veterinary Pediatrics Dogs and Cats from Birth to Six Months* Ch. 4 (W.B. Saunders, 2001).

[21] Meloni T (2014). A survey on bacterial involvement in neonatal mortality in dogs. Vet. Ital..

[22] Dahlinger J, Marks SL, Hirsh DC (1997). Prevalence and identity of translocating bacteria in healthy dogs. J. Vet. Intern. Med..

[23] Ghosh A (2013). Mortality in kittens is associated with a shift in ileum mucosa-associated enterococci from Enterococcus hirae to biofilm-forming Enterococcus faecalis and adherent Escherichia coli. J. Clin. Microbiol..

[24] Huda S, Chaudhery S, Ibrahim H, Pramanik A (2014). Neonatal necrotizing enterocolitis: clinical challenges, pathophysiology and management. Pathophysiology.

[25] Elgin TG, Kern SL, McElroy SJ (2016). Development of the neonatal intestinal microbiome and its association with necrotizing enterocolitis. Clin. Ther..

[26] Pitlik SD, Koren O (2017). How holobionts get sick-toward a unifying scheme of disease. Microbiome.

[27] Spor A, Koren O, Ley R (2011). Unravelling the effects of the environment and host genotype on the gut microbiome. Nat. Rev. Microbiol..

[28] Insoft RM, Sanderson IR, Walker WA (1996). Development of immune function in the intestine and its role in neonatal diseases. Pediatr. Clin. North Am..

[29] Murgas Torrazza R, Neu J (2011). The developing intestinal microbiome and its relationship to health and disease in the neonate. J. Perinatol..

[30] Dominguez-Bello MG (2010). Delivery mode shapes the acquisition and structure of the initial microbiota across multiple body habitats in newborns. Proc. Natl Acad. Sci. USA.

[31] McCracken VJ, Lorenz RG (2001). The gastrointestinal ecosystem: a precarious alliance among epithelium, immunity and microbiota. Cell. Microbiol..

[32] Nuriel-Ohayon M, Neuman H, Koren O (2016). Microbial changes during pregnancy, birth, and infancy. Front. Microbiol..

[33] Torres J (2020). Infants born to mothers with IBD present with altered gut microbiome that transfers abnormalities of the adaptive immune system to germ-free mice. Gut.

[34] Vuillermin PJ (2017). The maternal microbiome during pregnancy and allergic disease in the offspring. Semin. Immunopathol..

[35] Koren O (2012). Host remodeling of the gut microbiome and metabolic changes during pregnancy. Cell.

[36] Nuriel-Ohayon M (2019). Progesterone increases bifidobacterium relative abundance during late pregnancy. Cell Rep..

[37] Arrieta MC, Stiemsma LT, Amenyogbe N, Brown EM, Finlay B (2014). The intestinal microbiome in early life: health and disease. Front. Immunol..

[38] Penders J (2006). Factors influencing the composition of the intestinal microbiota in early infancy. Pediatrics.

[39] Mueller NT, Bakacs E, Combellick J, Grigoryan Z, Dominguez-Bello MG (2015). The infant microbiome development: mom matters. Trends Mol. Med..

[40] Uzan-Yulzari A (2021). Neonatal antibiotic exposure impairs child growth during the first six years of life by perturbing intestinal microbial colonization. Nat. Commun..

[41] Kuperman AA, Koren O (2016). Antibiotic use during pregnancy: how bad is it?. BMC Med..

[42] Calatayud M, Koren O, Collado MC (2019). Maternal microbiome and metabolic health program microbiome development and health of the offspring. Trends Endocrinol. Metab..

[43] Turjeman, S., Collado, M. C. & Koren, O. The gut microbiome in pregnancy and pregnancy complications. *Curr. Opin. Endocr. Metab. Res.*10.1016/j.coemr.2021.03.004 (2021).

[44] Guard BC (2017). Characterization of the fecal microbiome during neonatal and early pediatric development in puppies. PLoS ONE.

[45] Blake, A. B. et al. Developmental stages in microbiota, bile acids, and clostridial species in healthy puppies. *J. Vet. Intern. Med*. 10.1111/jvim.15928 (2020). 10.1111/jvim.15928 PMC7694855 33047396

[46] Yatsunenko T (2012). Human gut microbiome viewed across age and geography. Nature.

[47] Deusch O (2015). A longitudinal study of the feline faecal microbiome identifies changes into early adulthood irrespective of sexual development. PLoS ONE.

[48] Palmer C, Bik EM, DiGiulio DB, Relman DA, Brown PO (2007). Development of the human infant intestinal microbiota. PLoS Biol..

[49] Zakosek Pipan M, Kajdic L, Kalin A, Plavec T, Zdovc I (2020). Do newborn puppies have their own microbiota at birth? Influence of type of birth on newborn puppy microbiota. Theriogenology.

[50] Parra-Llorca A (2018). Preterm gut microbiome depending on feeding type: significance of donor human milk. Front. Microbiol..

[51] Hooda S, Vester Boler BM, Kerr KR, Dowd SE, Swanson KS (2013). The gut microbiome of kittens is affected by dietary protein:carbohydrate ratio and associated with blood metabolite and hormone concentrations. Br. J. Nutr..

[52] Muegge BD (2011). Diet drives convergence in gut microbiome functions across mammalian phylogeny and within humans. Science.

[53] Eggesbo M (2011). Development of gut microbiota in infants not exposed to medical interventions. APMIS.

[54] Koenig JE (2011). Succession of microbial consortia in the developing infant gut microbiome. Proc. Natl Acad. Sci. USA.

[55] Macke L, Schulz C, Koletzko L, Malfertheiner P (2020). Systematic review: the effects of proton pump inhibitors on the microbiome of the digestive tract-evidence from next-generation sequencing studies. Aliment. Pharmacol. Ther..

[56] Atarashi K (2017). Ectopic colonization of oral bacteria in the intestine drives TH1 cell induction and inflammation. Science.

[57] Harris KG, Chang EB (2018). The intestinal microbiota in the pathogenesis of inflammatory bowel diseases: new insights into complex disease. Clin. Sci..

[58] AS B (1986). A review of the fading puppy syndrome (also known as fading puppy complex). Vet. Annu..

[59] McIntyre A, Gibson PR, Young GP (1993). Butyrate production from dietary fibre and protection against large bowel cancer in a rat model. Gut.

[60] Buffie CG, Pamer EG (2013). Microbiota-mediated colonization resistance against intestinal pathogens. Nat. Rev. Immunol..

[61] Nixon SL, Rose L, Muller AT (2019). Efficacy of an orally administered anti-diarrheal probiotic paste (Pro-Kolin Advanced) in dogs with acute diarrhea: A randomized, placebo-controlled, double-blinded clinical study. J. Vet. Intern. Med..

[62] Bell JA (2008). Ecological characterization of the colonic microbiota of normal and diarrheic dogs. Interdiscip. Perspect. Infect. Dis..

[63] Lucena R, Olmedilla AB, Blanco B, Novales M, Ginel PJ (2018). Effect of Enterococcus faecium SF68 on serum cobalamin and folate concentrations in healthy dogs. J. Small Anim. Pract..

[64] Korpela K (2018). Intestinal microbiota development and gestational age in preterm neonates. Sci. Rep..

[65] Wandro, S. et al. The microbiome and metabolome of preterm infant stool are personalized and not driven by health outcomes, including necrotizing enterocolitis and late-onset sepsis. *mSphere*10.1128/mSphere.00104-18 (2018). 10.1128/mSphere.00104-18 PMC5990886 29875143

[66] Pammi M (2017). Intestinal dysbiosis in preterm infants preceding necrotizing enterocolitis: a systematic review and meta-analysis. Microbiome.

[67] Barko PC, McMichael MA, Swanson KS, Williams DA (2018). The gastrointestinal microbiome: a review. J. Vet. Intern. Med..

[68] Stockinger S, Hornef MW, Chassin C (2011). Establishment of intestinal homeostasis during the neonatal period. Cell Mol. Life Sci..

[69] Andersen AC (1957). Puppy production to the weaning age. J. Am. Vet. Med. Assoc..

[70] Potkay S, Bacher JD (1977). Morbidity and mortality in a closed foxhound breeding colony. Lab Anim. Sci..

[71] Suchodolski JS (2009). The effect of the macrolide antibiotic tylosin on microbial diversity in the canine small intestine as demonstrated by massive parallel 16S rRNA gene sequencing. BMC Microbiol..

[72] Deusch O (2014). Deep Illumina-based shotgun sequencing reveals dietary effects on the structure and function of the fecal microbiome of growing kittens. PLoS ONE.

[73] Shouval R (2020). Patterns of salivary microbiota injury and oral mucositis in recipients of allogeneic hematopoietic stem cell transplantation. Blood Adv..

[74] Bolyen E (2019). Reproducible, interactive, scalable and extensible microbiome data science using QIIME 2. Nat. Biotechnol..

[75] DeSantis TZ (2006). Greengenes, a chimera-checked 16S rRNA gene database and workbench compatible with ARB. Appl. Environ. Microbiol..

[76] Callahan BJ (2016). DADA2: High-resolution sample inference from Illumina amplicon data. Nat. Methods.

[77] Reitmeier S (2020). Arrhythmic gut microbiome signatures predict risk of type 2 diabetes. Cell Host Microbe.

