# Supplementary material for: Developmental intestinal microbiome alterations in canine fading puppy syndrome: a prospective observational study
Source: NPJ Biofilms Microbiomes. 2021 Jun 23;7:52. doi: 10.1038/s41522-021-00222-7 (PMC8222291; doi:10.1038/s41522-021-00222-7)
Supplement: Supplementary file 1 — Supplementary Information [file 41522_2021_222_MOESM1_ESM.pdf]

Supplementary Table 1: Rectal swab samples analyzed for microbial composition and diversity in healthy and fading puppies[*]

	Healthy puppies		Puppies later developing FPS		Total number of analyzed rectal swab samples
	Day-1	Day-8	Day-1	Day-8	
Microbial composition analysis	35	25	20	13	93
Rarefied datasets[1]	27	20	**13**	**8**	**68**

[*], rectal swab samples were obtained from 63/125 puppies, including 20 puppies with FPS, 31 healthy littermates of fading puppies and 12 puppies from unaffected litters; [1], Alpha- and beta- diversities were calculated based on rarefied datasets of **68** samples that passed quality control.

Supplementary Table 2: List of differentially abundant (ALDEx2) bacterial taxa of rectal microbiota in healthy neonatal puppies in Days 1 and 8 post-partum.

	A	B	C	D	E	F	G	H	I	J	K	L	M	N
1		we.ep	we.eBH	wi.ep	wi.eBH	rab.all	rab.win.Day_1	rab.win.Day_8	diff.btw	diff.win	effect	effect.low	effect.high	overlap
2	genus Epulopiscium	0.004015507	0.030045446	0.002654331	0.028816624	1.47318235	4.453458774	-0.573717218	-5.155154046	5.845039999	-0.827267834	-7.881157505	2.354995792	0.203008059
3	Clostridium celatum	0.000164206	0.007997055	7.07944E-05	0.00404868	3.940113447	5.893232809	0.328051379	-5.720564422	5.529037216	-0.958644808	-6.918251854	1.479694667	0.157895494
4	Clostridium perfringens	0.001492583	0.029084411	0.001071031	0.025695842	6.634315122	7.949813276	2.414506151	-4.693198433	5.39559767	-0.798391868	-7.871968397	1.316087569	0.209738339

Supplementary Table 3: List of differentially abundant (ALDEx2) bacterial taxa of rectal microbiota in FPS puppies in Days 1 and 8 post-partum.

	A	B	C	D	E	F	G	H	I	J	K	L	M	N
1		we.ep	we.eBH	wi.ep	wi.eBH	rab.all	rab.win.Day_1	rab.win.Day_8	diff.btw	diff.win	effect	effect.low	effect.high	overlap
2	Streptococcus luteciae	0.000365135	0.021682774	0.002885014	0.069509636	3.617225488	-0.012254677	8.698989193	8.56333469	6.246712952	1.089854538	-1.387253674	12.04939346	0.132748567

Supplementary Table 4: Top 10 most predictive (important) bacterial taxa for the random forest model.

Feature ID	FPS	Healthy	Sequence Length	Sequence
Pasteurellaceae	0.0043 [0.0005;0.0418]	0 [0;0]	187	TACGGAGGGTGCGAGCGTTAATCGGAATAACTGGGCGTAAAGGGCACGCAGGCGGTGACTTAAGTGAGGTGTGAAATCCCCGGGCTTAACCTGGGAATTGCATTTCAGACTGGGTCGCTAGAGTACTTTAGGGAGGGGTAGAATTCCACGTGTAGCGGTGAAATGCGTAGAGATGTGGAGGAATACCGAAGGCGAAGGCAGCCCCTTGGG
Clostridium celatum	0.0008 [0;0,029]	0.009 [0.002;0.042]	187	TACGTAGGTGGCGAGCGTTGTCCGGATTTACTGGGCGTAAAGGGAGCGTAGGCGGACTTTTAAGTGAGATGTGAAATACCCGGGCTCAACTTGGGTGCTGCATTTCAAACTGGAAGTCTAGAGTGCAGGAGAGGAGAATGGAATTCCTAGTGTAGCGGTGAAATGCGTAGAGATTAGGAAGAACACCAGTGGCGAAGGCGATTCTCTGGA
Ruminococcus gnavus	0 [0;0.0008]	0 [0;0.02]	187	TACGTAGGGGGCAAGCGTTATCCGGATTTACTGGGTGTAAAGGGAGCGTAGACGGCATGGCAAGCCAGATGTGAAAGCCCGGGGCTCAACCCCGGGACTGCATTTGGAACTGTCAGGCTAGAGTGTCGGAGAGGAAAGCGGAATTCCTAGTGTAGCGGTGAAATGCGTAGATATTAGGAGGAACACCAGTGGCGAAGGCGGCTTTCTGGA
Enterococcus	0.003 [0.001;0.047]	0.01 [0.004;0.027]	187	TACGTAGGTGGCAAGCGTTGTCCGGATTTATTGGGCGTAAAGCGAGCGCAGGCGGTTTCTTAAGTCTGATGTGAAAGCCCCCGGCTCAACCGGGGAGGGTCATTGGAAACTGGGAGACTTGAGTGCAGAAGAGGAGAGTGGAATTCCATGTGTAGCGGTGAAATGCGTAGATATATGGAGGAACACCAGTGGCGAAGGCGGCTCTCTGGT
Clostridium perfringens	0.002 [0;0.030]	0.04 [0.01;0.08]	187	TACGTAGGTGGCGAGCGTTATCCGGATTTACTGGGCGTAAAGGGAGCGTAGGCGGATGATTAAGTGGGATGTGAAATACCCGGGCTCAACTTGGGTGCTGCATTCCAAACTGGTTATCTAGAGTGCAGGAGAGGAGAGTGGAATTCCTAGTGTAGCGGTGAAATGCGTAGAGATTAGGAAGAACACCAGTGGCGAAGGCGACTCTCTGGA
Enterobacteriaceae	0.59 [0.097;0.751]	0.59 [0.34;0.76]	187	TACGGAGGGTGCAAGCGTTAATCGGAATTACTGGGCGTAAAGCGCACGCAGGCGGTTTGTTAAGTCAGATGTGAAATCCCCGGGCTCAACCTGGGAACTGCATCTGATACTGGCAAGCTTGAGTCTCGTAGAGGGGGGTAGAATTCCAGGTGTAGCGGTGAAATGCGTAGAGATCTGGAGGAATACCGGTGGCGAAGGCGGCCCCCTGGA
Gemella	0.0003 [0;0.001]	0	187	TACGTAGGTGGCAAGCGTTGTCCGGAATTATTGGGCGTAAAGCGCGCGCAGGTGGTTTAAAAAGTCTGATGTGAAAGCCCACGGCTCAACCGTGGAAGGTCATTGGAAACTGTTAAACTTGAGTGCAGGAGAGAAAAGTGGAATTCCTAGTGTAGCGGTGAAATGCGTAGAGATTAGGAGGAACACCAGTGGCGAAGGCGGCTTTTTGGC
Streptococcus	0.0003 [0;0.002]	0	187	TACGTAGGTCCCGAGCGTTGTCCGGATTTATTGGGCGTAAAGCGAGCGCAGGCGGTTTGATAAGTCTGAAGTAAAAGGCTGTGGCTTAACCATAGTATGCTTTGGAAACTGTTAAACTTGAGTGCAGAAGGGGAGAGTGGAATTCCATGTGTAGCGGTGAAATGCGTAGATATATGGAGGAACACCGGTGGCGAAAGCGGCTCTCTGGTC
Rothia nasimurium	0.0003 [0;0.001]	0	187	TACGTAGGGCGCGAGCGTTGTCCGGAATTATTGGGCGTAAAGAGCTTGTAGGCGGTTTGTCGCGTCTGCTGTGAAAGCCCGGGGCTTAACCCCGGGTTTGCAGTGGGTACGGGCTAACTAGAGTGCAGTAGGGGAGACTGGAATTCCTGGTGTAGCGGTGAAATGCGCAGATATCAGGAGGAACACCAATGGCGAAGGCAGGTCTCTGGG
S24-7	0.0003 [0;0.005]	0.0008 [0;0.00149]	187	TACGGAGGATGCGAGCGTTATCCGGATTTATTGGGTTTAAAGGGTGCGTAGGCGGCCTGCCAAGTCAGCGGTAAAATTGCGGGGCTCAACCCCGTACAGCCGTTGAAACTGCCGGGCTCGAGTGGGCGAGAAGTATGCGGAATGCGTGGTGTAGCGGTGAAATGCATAGATATCACGCAGAACCCCGATTGCGAAGGCAGCATACCGGCG

Relative frequency (abundance) and sequences of the top predictive amplicon sequencing variants by group. Data represented as median [0.25;0.75].

Supplementary Figure 1: Sequencing depth statistics (rarefaction curves).

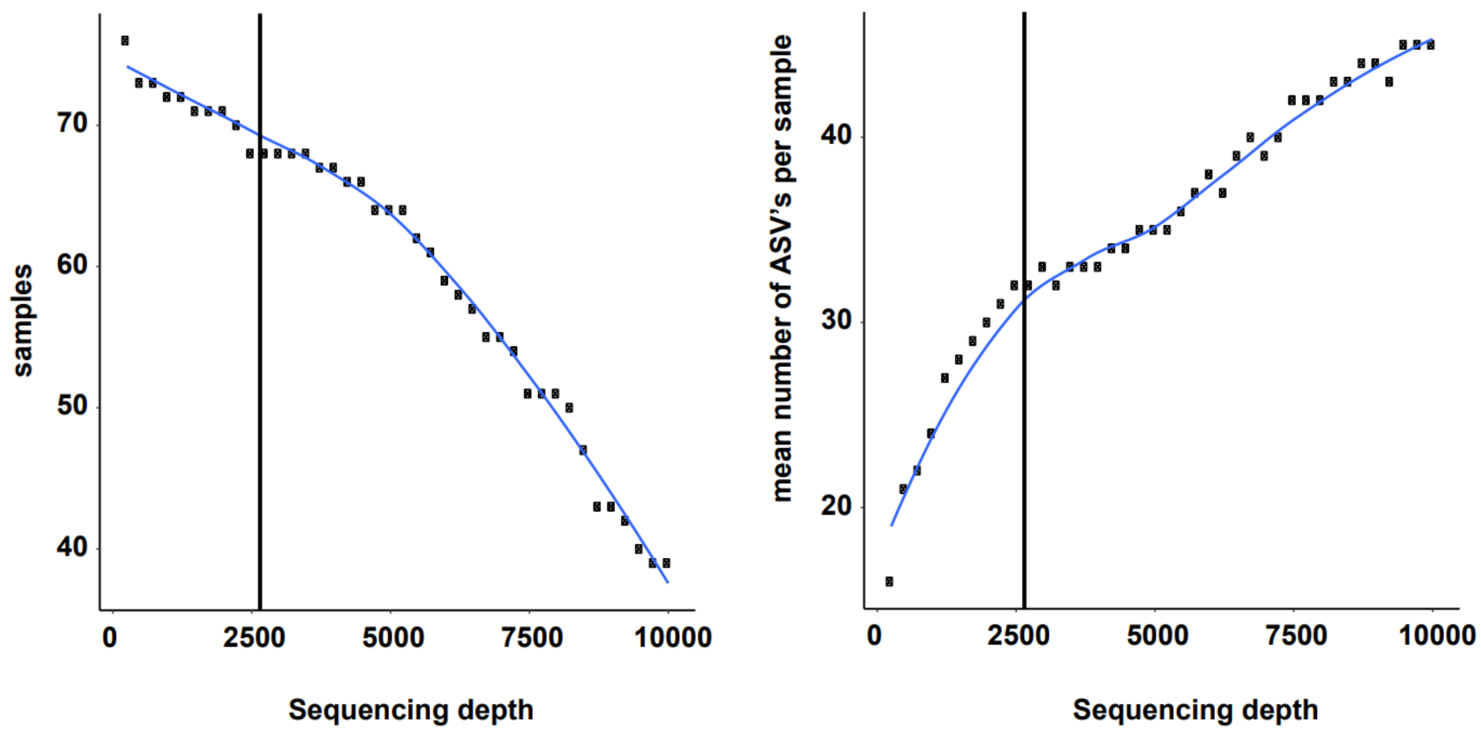

(a) The Y axis indicates the number of samples left after rarefaction to a certain sequencing depth (X axis). Vertical black line - rarefaction depth used in the study (2,649). **(b)** The Y axis indicates the mean number of observed features (amplicon sequencing variants) per sample after rarefaction to a certain sequencing depth (X axis). Vertical black line - rarefaction depth used in the study (2,649). Mean±sd read count across samples: 16000 ± 20589.
